# An Undiagnosed Paraganglioma in a 58-Year-Old Female Who Underwent Tumor Resection

**DOI:** 10.1155/2017/5796409

**Published:** 2017-10-15

**Authors:** William C. Fox, Matthew Read, Richard E. Moon, Eugene W. Moretti, Brian J. Colin

**Affiliations:** ^1^Department of Anesthesiology, Duke University Medical Center, Box 3823, Durham, NC 27710, USA; ^2^Critical Care Medicine, Department of Anesthesiology, Duke University Medical Center, Box 3823, Durham, NC 27710, USA; ^3^General, Vascular, Transplant Division, Department of Anesthesiology, Duke University Medical Center, Box 3823 Durham, NC 27710, USA

## Abstract

Paragangliomas and pheochromocytomas are rare neuroendocrine tumors that can have high morbidity and mortality if undiagnosed. Here we report a case of an undiagnosed paraganglioma in a 58-year-old female who underwent tumor resection. The patient became severely hypertensive intraoperatively with paroxysmal swings in blood pressure and then later became acutely hypotensive after tumor removal. She was managed in the surgical intensive care unit (SICU) postoperatively and discharged from the hospital without acute complications. We briefly discuss the epidemiology, clinical presentation, perioperative management, and possible complications of these tumors to assist healthcare providers if one were to encounter them.

## 1. Introduction

**Figure 1:**
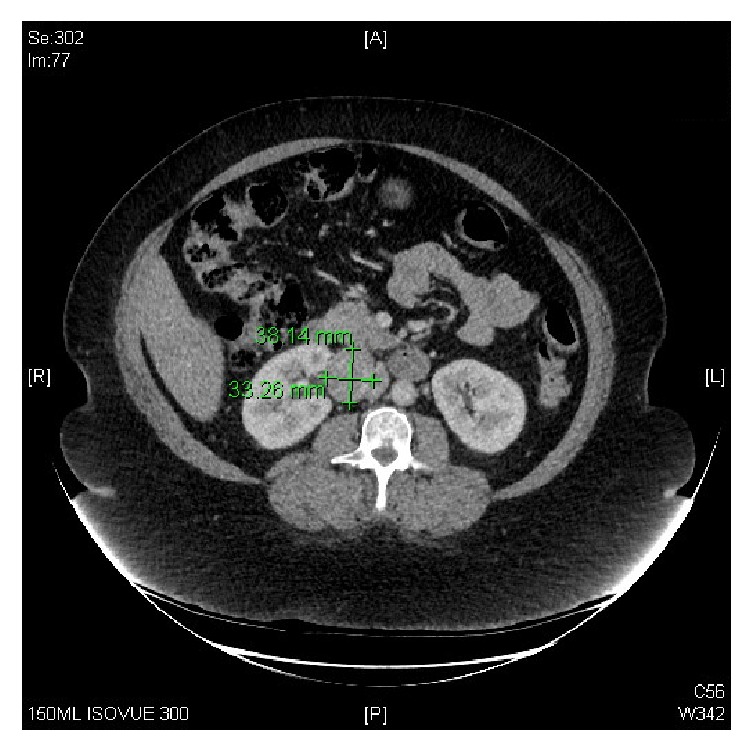
CT abdomen/pelvis with contrast.

Catecholamine-secreting tumors, also known as paragangliomas and pheochromocytomas, are rare neuroendocrine tumors that can have high morbidity and mortality if undiagnosed. Here we present a rare but interesting case of a resection of an undiagnosed paraganglioma in a 58-year-old African-American female. Her past medical history included hypertension, hyperlipidemia, obstructive sleep apnea on CPAP, asthma and COPD, morbid obesity, history of myocardial infarction in her 30s, and report of uncharacterized congestive heart failure and cerebrovascular accident in her early 50s with residual left-sided deficit. She presented for robotic-assisted laparoscopy for resection of an incidentally found right retroperitoneal mass. Two prior CT-guided fine needle aspiration biopsies of the mass were attempted but insufficient for diagnosis. Contrast-enhanced abdomen/pelvis CT revealed a 3.8 × 3.3 cm soft tissue mass at the right renal hilum abutting the inferior aspect of the right renal vein and right aspect of the inferior vena cava (Figure 1). Her home medications included aspirin, amlodipine-benazepril, furosemide, hydralazine, nebivolol, and spironolactone, as well as gabapentin, ibuprofen, and hydrocodone-acetaminophen for analgesia, and an albuterol inhaler and montelukast for asthma/COPD. Epidemiology, clinical presentation, perioperative management, and possible complications of these tumors are discussed.

## 2. Case Report

On the morning of surgery, the patient was hemodynamically stable. She received 2 mg IV midazolam preoperatively then underwent induction of general anesthesia with 100 mg IV lidocaine, 250 mcg IV fentanyl, 150 mg IV propofol, and 140 mg IV succinylcholine followed by 40 mg IV rocuronium after endotracheal tube placement. Shortly after incision and insufflation, she became hypertensive. Serial arterial BP readings were close to 250/140 mmHg with HR 60–70 bpm. Despite deepening the anesthetic gas and administering adequate doses of IV opioids and muscle relaxant, the patient's blood pressure did not improve. A total of 65 mg IV labetalol and 20 mg IV hydralazine were administered over the course of an hour. There was intermittent improvement in blood pressure to as low as 113/80 mmHg that lasted for a few minutes before soon returning to 250/140 mmHg. This cycle repeated during the redosing of antihypertensive agents but adequate control of blood pressure could not be obtained.

A clevidipine bolus (0.5 mg) was administered, followed by infusion at 1 mg/hr. Blood pressure remained close to 160/80 mmHg during the infusion but the patient became acutely tachycardic with HR 130 bpm, so the infusion was paused, resulting in an increase in BP to 257/163 mmHg. An esmolol bolus (100 mg) was administered, followed by an infusion which was started at 50 mcg/kg/min and clevidipine restarted once heart rate was controlled. Doses were escalated to 75 mcg/kg/min and 3 mg/hr, respectively, with BP ranging between 120/80 and 200/120 mmHg. Heart rate decreased to 90–100 bpm.

The procedure was converted from laparoscopic to open given the difficulty of resection due to the tumor's anatomy and patient instability. Her vital signs briefly appeared to “normalize” with BP 120/80 mmHg and HR 90 bpm before tumor removal so both clevidipine and esmolol infusions were discontinued. However, within several minutes of tumor removal, the patient became acutely hypotensive with systolic BP 60 mmHg and HR 80–90 bpm. A total of 400 mcg IV phenylephrine boluses were administered but the BP dropped to as low as 58/40 mmHg. Next, a total of 300 mcg of IV epinephrine was administered over several doses and the patient showed an improvement in blood pressure to 77/48 mmHg. At that time, the pathologist communicated that the tumor was a paraganglioma/pheochromocytoma. Norepinephrine and vasopressin infusions were started at 0.1 mcg/kg/min and 0.04 units/min, respectively. The patient was also given 100 mg IV hydrocortisone. Intraoperative transesophageal echocardiography (TEE) showed a hyperdynamic left ventricle with near collapse during systole and no wall motion abnormalities. The patient had received a total of 4300 ml of crystalloid and 1000 ml of colloid resuscitation intraoperatively prior to TEE.

She was taken to the SICU intubated on both pressors with ongoing fluid resuscitation. 3700 ml of crystalloid was administered postoperatively. The ICU team extubated and weaned her off pressors by postop day 1. CT brain without contrast did not show any acute intracranial abnormalities and cardiac enzymes were normal. She was discharged from the ward on postop day 4.

## 3. Discussion

Paragangliomas are rare neuroendocrine tumors that arise from the extra-adrenal autonomic paraganglia. These are small organs consisting mainly of neuroendocrine cells that are derived from the embryonic neural crest and have the ability to secrete catecholamines [1]. Paragangliomas are closely related to pheochromocytomas, which are sometimes referred to as intra-adrenal paragangliomas. The combined estimated annual incidence is approximately 0.8 per 100,000 person years [2]. Most are sporadic, but approximately one-third to one-half are associated with an inherited syndrome. The four genetic syndromes that are associated are multiple endocrine neoplasia 2A and 2B, neurofibromatosis type 1, von Hippel Lindau, and Carney-Stratakis syndrome. The male-to-female ratio is approximately equal among patients with hereditary paraganglioma, while sporadic tumors are more common in women than men (71 versus 29 percent) [3]. Most paragangliomas are diagnosed between third and fifth decade of life.

Paragangliomas can derive from parasympathetic or sympathetic paraganglia with similar frequencies. The majority of sympathetic paragangliomas arise outside of the skull base and neck anywhere along the sympathetic chain. About 75 percent arise in the abdomen, most often at the junction of the inferior vena cava and left renal vein. About 10 percent arise in the thorax. They excrete excess amounts of catecholamine (86 percent in one series) [4], usually almost always norepinephrine.

Manifestations of catecholamine-secreting tumors include hypertension, often paroxysmal, and are associated with episodic headache, sweating, and palpitations. Less commonly there may be orthostatic hypotension, weight loss, hyperglycemia, polyuria, polydipsia, visual blurring, papilledema, or constipation. The patient in this case mentioned postoperatively that she had previous episodes of periodic facial flushing, palpitations, and intermittent chest pain. Given her history of myocardial infarction and cerebrovascular accident, these may have been attributed to her paraganglioma. Also, it is difficult to rule out an adrenal or extra-adrenal tumor based on her CT images. Hence, this should have alerted her physicians of a possible paraganglioma. Although she had two unsuccessful fine needle biopsies, preoperative labs including plasma and urinary metanephrines and catecholamines may have suggested a possible catecholamine-secreting tumor.

Undiagnosed pheochromocytoma has a high perioperative mortality risk, as great as 80 percent perioperatively according to one report [5] and 27 percent in a different series [6]. However, it is hypothesized that, with advancement in perioperative care and optimal management of these tumors as discussed below, the mortality is lower compared to 30 years ago. Patients can become hypertensive during induction, positioning, or tumor resection. Chronic catecholamine excess causes hypovolemia and patients can become severely hypotensive, as in this case, if adequate volume resuscitation is not performed. It is prudent to evaluate for adverse events following hypertensive and hypotensive episodes. Serial neurological evaluations, electrocardiograms, and cardiac enzymes should be considered as part of management. Plasma catecholamine concentrations should return to normal after about a week from tumor resection.

Complications of surgery are primarily due to severe preoperative hypertension, high secretion tumors, or repeat intervention for recurrence. In one study, adverse perioperative events occurred in 32 percent of cases [7]. The most common adverse event was sustained hypertension in 25 percent of the patients. There were no perioperative deaths, myocardial infarctions, or cerebrovascular events. Despite premedication of most patients with phenoxybenzamine and a beta-blocker, varying degrees of intraoperative hemodynamic lability occurred.

In preparation for surgery, preoperative medical therapy should focus on controlling blood pressure and volume resuscitation. An alpha-blocker, preferably phenoxybenzamine is started 10–14 days prior to procedure. Beta blockade may be necessary for control over arrhythmias or tachycardia. It should never be started prior to alpha blockade because blockade of vasodilatory peripheral beta-adrenergic receptors with unopposed alpha-adrenergic receptor stimulation can lead to hypertensive crisis.

Hypertensive crisis may occur intraoperatively despite optimum medical therapy due to various stimuli. A retrospective study at a single institution of 73 patients undergoing resection for pheochromocytoma found higher preoperative plasma norepinephrine concentration, larger tumor size (>4 cm), and more pronounced postural blood pressure fall after alpha blockade (>10 mmHg) correlated with intraoperative hypertensive events [8].

Treatment for hypertensive crisis has included intravenous sodium nitroprusside, phentolamine, or nicardipine infusions. Cardiac arrhythmias can be managed with IV lidocaine bolus or esmolol infusion (extreme caution is necessary when administering a beta blockade without concurrent or preexisting alpha blockade). Magnesium sulfate has also been shown to inhibit catecholamine release. It is a calcium channel antagonist of vascular smooth muscle, which acts to decrease intracellular calcium. One major effect of decreased intracellular calcium would be inactivation of calmodulin-dependent myosin light chain kinase activity and decreased contraction, causing smooth muscle relaxation [9]. Magnesium sulfate appears to be predominantly an arterial vasodilator, reducing peripheral resistance but with minimal effects on venous return or pulmonary capillary wedge pressure. A total of 10–20 grams of magnesium sulfate may be required perioperatively to achieve blood pressure control. One series was successful in preventing catecholamine surge by keeping the plasma magnesium concentration between 2.5 and 3 mmol/L (6.1–7.3 mg/dL), requiring a magnesium infusion of 1-2 grams per hour [10].

Medications that produce histamine release such as morphine and medications that inhibit catecholamine reuptake or cause indirect increase of catecholamines, such as droperidol, ketamine, ephedrine, and metoclopramide, should all be avoided.

In summary, this case illustrates the importance of obtaining a detailed clinical history and evaluation if there is a high suspicion for paraganglioma/pheochromocytoma. Although not all patients with hypertension and an incidentally found adrenal or extra-adrenal tumor on imaging may have a catecholamine-secreting tumor, plasma and urinary metanephrines and catecholamines may assist in the diagnosis of one, especially if there is a history of hypertensive crisis, paroxysmal swings in blood pressure, history of myocardial infarction or cerebrovascular accident, or any other manifestation of catecholamine-secreting tumors. Optimal medical management such as starting patients on alpha-adrenergic antagonists weeks prior to any elective procedure and discussing with the patient perioperative complications and risks are essential. From intraoperative TEE findings, we found that postresection hypotension is almost always associated with hypovolemia from chronic catecholamine secretion and should be treated by aggressive fluid resuscitation. Serial neurological and cardiac evaluations should be considered as part of management in a patient with severe intraoperative hypertension or hypotension.
